# A randomised controlled feasibility study of food-related computerised attention training versus mindfulness training and waiting-list control for adults with overweight or obesity: the FOCUS study

**DOI:** 10.1186/s40337-023-00780-5

**Published:** 2023-04-12

**Authors:** Daniela Mercado, Jessica Werthmann, Tiago Antunes-Duarte, Iain C. Campbell, Ulrike Schmidt

**Affiliations:** 1 Section of Eating Disorders, Department of Psychological Medicine, Institute of Psychiatry, Psychology and Neuroscience, King’s College London, London, UK; 2 Department of Clinical Psychology and Psychotherapy, Institute of Psychology, Albert-Ludwigs University of Freiburg, Freiburg im Breisgau, Germany; 3 Hospital de Santa Maria, Centro Hospitalar Universitário Lisboa Norte, EPE, Lisbon, Portugal; 4 South London and Maudsley NHS Foundation Trust, London, UK

**Keywords:** Obesity, BED, Attention training, Mindfulness, Attention bias, Trial

## Abstract

**Background:**

In a feasibility randomised controlled trial in people with overweight/obesity with and without binge eating disorder (BED) symptoms, we assessed eight weekly sessions of attention bias modification training (ABMT) and mindfulness training (MT) versus waiting list (WL) and explored potential mechanisms.

**Methods:**

45 participants were randomly allocated to one of three trial arms. Primary outcomes were recruitment, retention and treatment adherence rates. Secondary outcomes included measures of eating behaviour, mood, attention and treatment acceptability. Assessments were conducted at baseline, post-intervention (week 8), and follow-up (week 12).

**Results:**

Participant retention at follow-up was 84.5% across groups. Session completion rates in the laboratory were 87% for ABMT and 94% for MT, but home practice was much poorer for ABMT. Changes in BMI and body composition were small between groups and there was a medium size BMI reduction in the MT group at follow-up. Effect sizes of eating disorder symptom changes were not greater for either intervention group compared to WL, but favoured ABMT compared to MT. Hedonic hunger and mindful eating scores favoured MT compared to ABMT and WL. ABMT reduced attention biases towards high-calorie food cues, which correlated with lower objective binge eating days at post-intervention. No significant changes were observed in the MT, or WL conditions.

**Conclusions:**

Both ABMT and MT have potential value as adjuncts in the treatment of obesity and BED, and a larger clinical trial appears feasible and indicated.

*Trial registration*: ISRCTN Registry, ISRCTN15745838. Registered on 22 May 2018.

**Supplementary Information:**

The online version contains supplementary material available at 10.1186/s40337-023-00780-5.

## Abstract (summary)

In this small research study, people who were classified as overweight or living with obesity (with or without symptoms of binge eating disorder) received either a mindfulness intervention, a “subconscious” attention-based intervention called attention bias modification training (ABMT) or were put on a waiting list for 8 weeks. People in the mindfulness group experienced improvements in emotional eating, mindful eating, and “impulsive” eating. Those who received the attention-based training paid less attention to highly caloric food after the intervention which correlated with fewer binge eating episodes. Results from this study suggest that both types of interventions have potential as add-on treatments for obesity and binge eating disorder, but larger studies are necessary to assess their clinical impact.

**Supplementary Information:**

The online version contains supplementary material available at 10.1186/s40337-023-00780-5.

## Background

There is a bi-directional relationship between obesity and BED [1]. For example, it is estimated that individuals with BED are 3–6 times more likely to be overweight or to have obesity than those without an eating disorder (ED) [2–4]. It is also estimated that around 30% of people with obesity who are seeking weight loss treatment have BED symptoms [5] and up to 47% of those seeking bariatric surgery meet a formal diagnosis of BED [6]. Furthermore, a high body mass index (BMI) in early adolescence is considered a key risk factor for disordered eating [7].

Common interventions for obesity (with or without BED) focusing only on lifestyle changes and dieting, tend not to have lasting effects [8–10], whereas interventions that combine lifestyle advice and psychological support appear more promising (e.g. HAPIFED[11]). Arguably, the lack of lasting effects in the widely used ‘lifestyle only’ interventions is because cognitive elements associated with overeating are not addressed: these include attention processes in general, and attention biases (AB) to food cues in particular. AB in obesity has been linked to sustained attention to high caloric (High-Cal) food cues [12–14], and is reported to contribute to food craving and overeating [15–17]. Interventions targeting attention processes in obesity and BED have provided encouraging results [18]. Examples are attention bias modification trainings (ABMT) [15, 19, 20] and mindfulness-based interventions (MBIs) (e.g. [21–23]). However, although these interventions target attention, they differ in their approach.

ABMT is a computerised training that aims to modify a relatively automatic attention process implicitly. While ABMT has been mainly used in the treatment of depression and anxiety disorders using disorder specific cues [24], a version involving food cues has also been investigated [13, 16].In the context of modifying AB to food, ABMT has been designed to train attention away from High-Cal food cues and/or towards healthy food cues. It has been hypothesised that this attentional shift will modify the valence of food in an implicit way (i.e. High-Cal food will become less rewarding and/or healthy food more appealing) [25], consequently affecting eating behaviour [19]. In line with this hypothesis, studies have shown that the subjective valence of food cues can be modified through a cue-approach behaviour using a go/no go training. In these experiments, there is an increase in the subjective value of the food cues paired with the “go” signal as a result of the trained approach response [26, 27]. Even though results have been mixed, preliminary results on the effects of ABMT are promising. Meta-analyses have shown medium effect sizes in reducing High-Cal food consumption after training participants to look away from High-Cal food cues using ABMT [28] and in modifying AB towards food cues [29]. A study conducted by Schmitz and Svaldi [18] showed a significant reduction in subjective food cravings after training people with BED to look away from food-cues using an ABMT. However, most studies involved participants with healthy weight, and had a single-session design [15, 19, 30]. To our knowledge, only two ABMT studies included people with obesity/BED and used a multi-session design [14, 31]. Boutelle et al. (2016) conducted a feasibility open trial using eight weekly sessions of ABMT in overweight people or people with obesity who binge eat, and reported positive results in reducing weight, eating disorder symptoms, binge eating and attention bias after training. However, this study had a small sample size and no control condition [31]. Kemps et al. (2016) employed five weekly sessions of ABMT to train a sample of women with a BMI > 25 kg/m^2^ to look away from (“avoid”) or towards (“attend”) food pictures and found an increase in AB to food cues in the “attend” group and a decrease in the “avoid” group [14]. However, both multi-session studies used a version of ABMT based on motor responses as opposed to eye movements, i.e., they focused on later stages of attention (it takes longer to create a motor response than to direct one´s eye gaze to an object in a relatively automatic way), and represents an indirect measure of AB [32, 33]. Moreover, the clinical potential of ABMT, as well as its credibility and acceptability, has not been tested in comparison to another active intervention within a randomised controlled trial (RCT).

MBIs train people to explicitly attend to the present moment in a non-judgmental way [34, 35]. The focus of sustained attention can vary depending on the technique, e.g., attention can be directed towards the breath, or towards inner body cues related to hunger and satiety. The latter is part of a mindfulness technique (“mindful eating”), which trains people to pay attention to the process of eating, from the sensory characteristics of food, to their physiological, psychological and emotional reactions to it [36]. Arguably, MBIs have the potential to modify AB to food by strengthening general attention control, which has been observed as enhanced activation of the anterior cingulate cortex (ACC) in non-expert meditators [37]. In turn, this may enable attentional disengagement from High-Cal food cues. Greater attention control as a consequence of MBIs can also increase emotion regulation through a reduced activation of the amygdala in response to salient stimuli (i.e., food) [38, 39], which could reduce problematic eating behaviour [40]. Several studies (including RCTs) have investigated the potential of MBIs in obesity and BED and have reported some improvements in weight and in eating behaviours including medium-large effect sizes for reducing binge eating [9, 40–46], emotional eating [9, 42, 43], impulsive eating [44], restrained eating [9] and external eating [43], and medium effect sizes in reducing body weight [9]. However, the effect of this controlled, or “top-down” approach on more automatic, or “bottom-up” processes such as AB to food cues, has not been investigated in the context of overeating.

Given the different approaches employed by ABMT and MBIs (implicit vs explicit training) in targeting a common substrate (attention), we have investigated the clinical effects, acceptability and credibility of these attention trainings on weight loss, eating behaviour, and AB for food. Exploring their potential mechanisms will help determine the most suitable adjunct treatment intervention for people with obesity with and without BED.

This study was a feasibility RCT comparing ABMT vs. a mindfulness training (MT), to a waitlist (WL) control group for people with overweight/obesity. The primary objective was to assess recruitment, retention rates, and treatment adherence (session completion; home practice). Secondary objectives were to:

- Estimate between-group effect sizes and standard deviations of clinical outcomes to inform future sample size calculations; estimate within-group effect sizes and standard deviations of clinical outcomes to assess change processes over time in each group.
- Assess credibility and acceptability of trial interventions.
- Measure change in attention bias as a potential underlying mechanism.

## Materials and methods

### Design and participants

The study was conducted in accord with the Consolidated Standards of Reporting Trials (CONSORT) for feasibility RCTs [47]. In a parallel group, randomised, trial participants were allocated to 8 weeks of either an attention bias modification training (ABMT), a mindfulness training (MT) or a waitlist control (WL). Participants were recruited through the university circular mail, posters, online adverts and from participation in previous research. Main inclusion criteria were: age 18 years or older and a BMI of 25 kg/m^2^ or above. Potential participants were screened to assess suitability for inclusion. Main exclusion criteria included: a current DSM-5 diagnosis of anorexia nervosa (AN), bulimia nervosa (BN) or other specified feeding or eating disorder (OSFED); diabetes mellitus; current regular mindfulness or meditation practice [48]. After providing informed written consent to taking part in the study, participants were allocated randomly by minimisation to one of three study arms (MT, ABMT or WL). Randomisation was carried out by an independent researcher not involved in the trial. Sample size calculations were based on the recommendation by Julious, 2005 for feasibility trials (n = 12 /arm) [49], and included a drop-out correction factor, assuming an attrition rate of 25% from baseline to follow-up. Given the heterogeneous nature of each intervention, blinding of participants or outcome assessors was not possible.

### Interventions

Food related versions of ABMT and MT were used. Full details are described in our protocol paper [48]. Briefly, in ABMT, participants were trained to direct their attention (eye gaze) towards healthy food cues and away from High-Cal food cues using an anti-saccade task and measuring eye movements [15]. The MT training was app-based and was provided by the company Headspace®: this training included a guided meditation, focusing on mindful eating and coping with cravings. In both groups, participants were offered 8 weekly sessions of in-person training (10 min each), interspersed with a recommendation to do once daily home practice of the same length. In-person ABMT training included recording of eye movements, while home practice was an online version of the training which only recorded training accuracy (i.e., not eye movements). For the MT, in-person, and home practice followed the same procedure of listening to the sequential mindfulness meditations in the Headspace® App. In the WL condition, participants completed assessments at baseline and 8 weeks and then were allowed to choose the training of their preference.

### Outcome measurement

Main feasibility outcomes were recruitment, study retention and treatment adherence rates. The latter consisted of session attendance to the laboratory and home practice. To judge whether to proceed with a future larger RCT, we pre-specified two criteria: recruitment as planned (i.e., reaching the desired sample size within 1 year from commencement) and retention rates from baseline to follow-up > 75%. Details on secondary clinical and neurocognitive outcomes are described in our protocol paper [48]. Outcome measures reported here are described below. All measures were completed at baseline and end of treatment (week 8) and the active intervention groups also completed an online self-report follow-up (week 12 post-randomisation).

#### Eating behaviour-related measures

These included body mass index (BMI), body composition (i.e. percentage of body fat) using a bioelectrical impedance scale (InBody S10), the Eating Disorder Examination Questionnaire, EDE-Q [50], the Power of Food Scale, PFS [51] assessing psychological impact of food cues and a Bogus taste test [52]. The Bogus taste test is a behavioural measure which assesses food consumption of highly palatable food (i.e. crisps, chocolates and soft sweets) after asking participants to rate the sensory characteristic of these food items for 10 min. The total food intake is calculated by measuring the difference in grams before and after the taste test.

#### Mood symptoms

These used the Depression, Anxiety and Stress- Scale (DAAS-21) [53] and the state-anxiety of the State and Trait Anxiety Inventory (STAI) [54].

#### Mindfulness-related measures

General mindfulness and mindful eating were assessed using the Mindful Awareness and Attention Scale (MAAS) [55] and the Mindful Eating Questionnaire (MEQ) [56].

#### Treatment acceptability and credibility

Questions related to acceptability, credibility and perceived benefits/drawbacks of the active interventions were administered at week 8 post-randomisation and included items like “How useful did you find this training?”. Training of choice after the waiting period for WL participants was also considered.

#### Attention bias for food cues

AB for food cues was assessed using a modified version of the dot-probe task with high- and low-caloric food pictures [12, 33, 57] while recording eye movements. We used initial fixation duration bias (i.e., the mean duration of initial fixation(s) directed towards food versus non-food cues before the initial gaze is shifted), reflecting early attention maintenance and duration bias (i.e., the mean duration of fixation time on food vs. non-food cues), indicating total sustained attention on High-Cal food cues, as indices for biased attention (i.e., AB) to High-Cal food cues [58]. Positive scores indicate initial and total attentional approach towards High-Cal food cues [59].

### Data analysis

Primary feasibility outcomes are presented as n/N (%). Post-intervention and follow-up group means and SDs for the clinical outcomes (8- and 12-weeks post-randomisation) were adjusted for baseline (i.e., change scores) and are presented with between-group effect sizes (Cohen’s *d*), alongside 95% confidence intervals (CI). Within-group effect sizes (Cohen's *d* as suggested by [60]) were also estimated between baseline and both, post-intervention and follow-up. To explore change in attention bias as a potential mechanism, group differences for attention bias indices (i.e., initial fixation duration bias and duration bias) were calculated using a mixed model ANOVA to test within-and between-group differences over time. Last observation carried forward imputation was used to deal with missing data.

## Results

### Primary feasibility outcomes

**Fig. 1:**
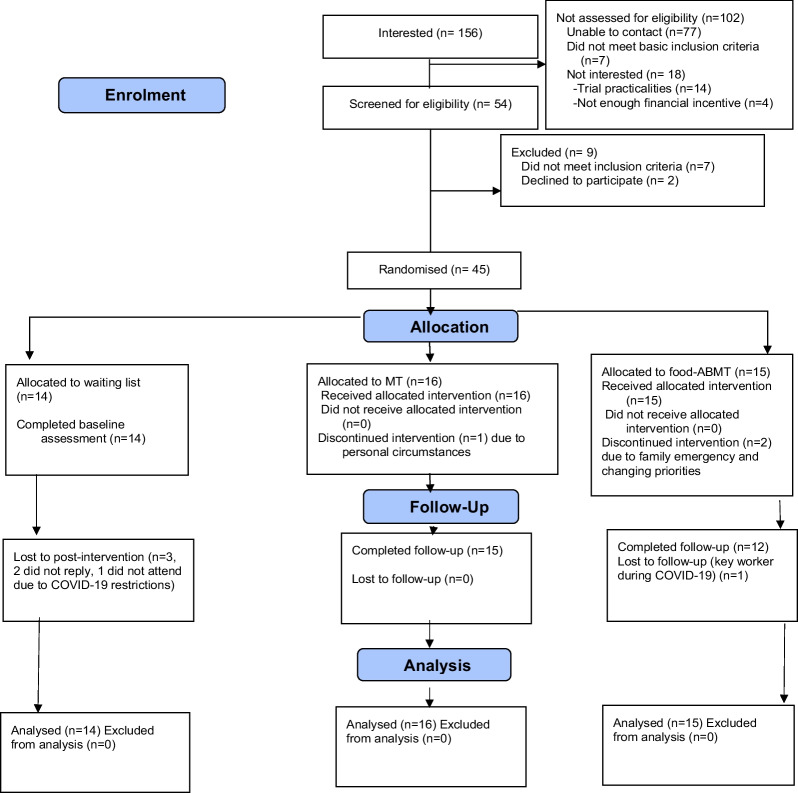
CONSORT flow diagram

Recruitment was completed within a predefined timeframe (February 2019-January 2020). For baseline characteristics, see Table 1, and for patient flow through the study, see Fig. 1. Of 156 people who expressed interest, 54 were screened and 45 (n = 34 female, n = 11 male) were randomly allocated to one of the trial arms (WL n = 14, MT n = 16, ABMT n = 15). Study retention rates were 86.7% (39/45) and 84.5% (38/45) at post-intervention and follow-up respectively. Session completion rates (defined as attending 8/8 sessions) were 87% and 94% respectively for the ABMT and MT groups. In the MT condition, one individual stopped the intervention after one training session. In the ABMT group, two individuals dropped-out after 3 and 2 sessions respectively.

**Table 1:** Baseline demographic and clinical characteristics

Demographic details	WL (n = 14)	MT (n = 16)	ABMT (n = 15)	Whole sample
Age (years) (median [IQR])	31 (10.75)	29 (29)	35 (10)	32 (13)
BMI (kg/m^2^) (mean ± SD)	35.4 (6.6)	33 (4.2)	34.8 (8.5)	34.3 (6.57)
Body fat percentage (mean ± SD)	34.2 (13.5)	39.7 (9.4)	38.8 (10.3)	37.7 (11.15)
BMI category (overweight/obesity) (n)	4/10	5/11	4/11	13/32
BED symptoms (yes/no) (%)	64/36	38/62	40/60	47/53
Ethnicity (Caucasian/BAME) (n)	5/9	10/6	10/5	25/20
Highest level of education (GCSE/AS and above)	1/13	0/16	0/15	1/44
Gender (male/female) (%)	29/71	19/81	20/80	22/78
Current diagnosis of psychiatric disorder (no/yes) (%)	64/36	77/23	73/27	71/29
Current weight loss program involvement (yes/no) (%)	14/86	0/100	13/87	9/91
EDE-Q Total score (mean ± SD) (WL n = 13, MT n = 14, ABMT n = 15)	2.8 (1.4)	2.3 (0.9)	2.8 (1.2)	2.6 (1.2)
EDE-Q binge episodes (mean ± SD) (days) (WL n = 13, MT n = 14, ABMT n = 15)	7.8 (7.9)	6.5 (7.3)	9 (8.7)	7.8 (7.91)
DASS-depression subscale (mean ± SD)	5.5 (4.6)	3.3 (2.1)	5 (4.2)	4.5 (3.82)
DASS-anxiety subscale (mean ± SD)	5.5 (4.4)	3.3 (1.9)	2.5 (2.9)	3.7 (1.0)
DASS-stress subscale (mean ± SD)	8.5 (4.8)	5.7 (3.7)	5.8 (3.6)	6.6 (4.21)
STAI-S (mean ± SD) (WL n = 13, MT n = 15, ABMT n = 15)	44.3 (9.5)	33.8 (5.8)	36.2 (10.5)	37.8 (9.7)
MAAS (mean ± SD) (WL n = 12, MT n = 14, ABMT n = 15)	3.9 (0.99)	3.6 (0.89)	3.6 (1.13)	3.7 (1.0)
MEQ-Total (mean ± SD) (WL n = 7, MT n = 6, ABMT n = 11)	2.6 (0.26)	2.1 (0.35)	2.3 (0.48)	2.3 (0.41)
MEQ-Awareness (mean ± SD) (WL n = 13, MT n = 16, ABMT n = 14)	2.7 (0.41)	2.5 (0.55)	2.1 (0.67)	2.4 (0.59)
MEQ-Distraction (mean ± SD) (WL n = 12, MT n = 16, ABMT n = 13)	2.5 (0.53)	2.6 (0.72)	2.5 (0.76)	2.5 (0.67)
MEQ-Disinhibition (mean ± SD) (WL n = 9, MT n = 15, ABMT n = 13)	2.3 (0.64)	1.7 (0.44)	2.1 (0.64)	2.0 (0.61)
MEQ-Emotional (mean ± SD) (WL n = 13, MT n = 15, ABMT n = 14)	2.5 (0.81)	2.1 (0.72)	2.2 (0.80)	2.32 (0.77)
MEQ-External (mean ± SD) (WL n = 12, MT n = 8, ABMT n = 12)	2.5 (0.38)	2.6 (0.51)	2.4 (0.42)	2.54 (0.42)
PFS (mean ± SD)	3.5 (0.85)	3.7 (0.87)	3.5 (0.85)	3.6 (0.84)
Bogus taste test (mean ± SD) (g)	79.8 (49.2)	84.4 (43.3)	79 (52.2)	81.2 (47.22)

*WL* waitlist, *MT* mindfulness training, *ABMT* Attention Bias Modification Training, *IQR* interquartile range, *n* number of observations, *BED* Binge Eating Disorder, *BAME* Black, Asian and minority ethnic, *BMI* body mass index, *EDE-Q* Eating Disorder Examination Questionnaire, *DASS* Depression, Anxiety, Stress scale, *STAI-S* State and Trait Anxiety Inventory, State version, *MASS* Mindfulness Attention and Awareness Scale, *MEQ* Mindful Eating Questionnaire, *PFS* Power of Food Scale

Daily home practice of training was recommended (6 days/week × 8 weeks = 48 home training sessions). MT participants completed the home practice 51/48 times on average versus 17/48 times on average in the ABMT group (i.e., 106% vs. 35% of recommended practice respectively).

### Secondary outcomes: baseline to post-intervention (week 8)

Means, standard deviations and effect sizes (including confidence intervals) of the clinical outcomes from baseline to post-intervention can be seen in Tables 2 and 3 for between-groups and within-groups respectively.

**Table 2:** Mean change scores (post-treatment scores adjusted for baseline) for the clinical outcome measures at 8-week post-randomisation timepoints including the number of participants (N), means, and standard deviations (SD) for each trial arm, and the estimated between-groups effect size (Cohen’s *d* with 95% confidence intervals)

Assessment	WL			MT			ABMT			Between- subject *d* (95% CI)		
	*N*	Mean	SD	*N*	Mean	SD	*N*	Mean	SD	WL versus MT	WL versus ABMT	MT versus ABMT
8-weeks post-randomisation (adjusted for baseline)												
BMI	11	− 0.38	0.75	15	− 0.06	0.94	13	− 0.13	0.80	− 0.36 (− 1.14 to 0.42)	− 0.31 (− 1.11 to 0.49)	− 0.07 (− 0.82 to 0.66)
Body fat percentage	11	5.8	15.4	14	0.19	5.13	10	2.92	8.18	0.51 (− 0.28 to 1.31)	0.23 (− 0.62 to 1.08)	0.41 (− 0.40 to 1.23)
EDE-Q Global	10	− 0.62	0.70	13	− 0.20	0.57	12	− 0.50	1.15	− 0.66 (− 1.50 to 0.19)	− 0.12 (− 0.96 to 0.71)	− 0.33 (− 1.12 to 0.45)
EDE-Q binge episodes	11	− 4.18	6.44	15	− 2.6	5.69	12	− 3.5	8.70	− 0.26 (− 1.04 to 0.52)	− 0.08 (− 0.90 to 0.73)	− 0.12 (− 0.88 to 0.63)
DASS-21 Depression	11	− 1.0	3.97	15	0.26	3.97	12	− 1.58	3.14	− 0.31 (− 1.0 to 0.46)	0.16 (− 0.65 to 0.98)	− 0.50 (− 1.27 to 0.26)
DASS-21 Anxiety	11	0.0	2.48	15	0.06	2.40	12	− 0.41	1.31	− 0.02 (− 0.80 to 0.75)	0.21 (− 0.61 to 1.03)	− 0.24 (− 1.0 to 0.52)
DASS-21 Stress	11	− 1.18	3.25	15	− 0.33	3.82	12	− 1.75	4.07	− 0.23 (− 1.01 to 0.54)	0.15 (− 0.66 to 0.97)	− 0.35 (− 1.12 to 0.40)
STAI-S	10	− 5.8	9.82	14	1.07	8.13	13	0.15	14.7	− 0.77 (− 1.60 to 0.07)	− 0.46 (− 1.29 to 0.37)	− 0.07 (− 0.83 to 0.67)
MAAS	8	− 0.15	0.45	13	0.27	0.76	12	0.37	1.01	− 0.62 (− 1.52 to 0.28)	− 0.62 (− 1.53 to 0.30)	0.11 (− 0.66 to 0.90)
MEQ Global	3	0.007	0.16	5	0.23	0.19	7	0.34	0.48	− 0.84 (− 2.31 to 0.69)	− 0.63 (− 2.0 to 0.77)	0.28 (− 0.87 to 1.43)
MEQ-Awareness	10	0.00	0.33	14	0.10	0.76	11	0.29	0.57	− 0.16 (− 0.97 to 0.65)	− 0.62 (− 1.49 to 0.26)	0.28 (− 0.51 to 1.0)
MEQ-Distraction	8	0.04	0.51	15	0.04	0.75	10	0.43	0.91	− 0.00 (− 0.86 to 0.85)	− 0.50 (− 1.44 to 0.44)	0.47 (− 0.34 to 1.28)
MEQ-Disinhibition	6	0.23	0.32	13	0.56	0.36	9	0.26	0.48	− 0.94 (− 1.94 to 0.08)	− 0.07 (− 1.10 to 0.96)	− 0.72 (− 1.59 to 0.16)
MEQ-Emotional	10	0.05	0.32	13	0.51	0.59	11	0.40	0.77	**− 0.94 (− 1.80 to − 0.06)**	− 0.59 (− 1.46 to 0.29)	− 0.16 (− 0.96 to 0.64)
MEQ-External	6	0.22	0.47	7	− 0.35	0.43	8	− 0.08	0.43	**1.27 (0.04 to 2.46)**	0.67 (− 0.43 to 1.75)	0.63 (− 0.42 to 1.66)
PFS	11	− 0.2	0.67	15	− 0.85	0.78	12	− 0.48	0.60	**0.88 (0.05 to 1.69)**	0.44 (− 0.38 to 1.26)	0.52 (− 0.25 to 1.28)
BOGUS test (g)	11	11.6	34.7	15	− 9	47.89	13	− 24.8	46.6	0.48 (− 0.31 to 1.26)	**0.87 (0.02 to 1.71)**	− 0.33 (− 1.07 to 0.41)

Post-intervention scores minus baseline scores. Bold font signifies that the CI do not include 0. *WL* waitlist, *MT* mindfulness training, *ABMT* Attention Bias Modification Training, *N* number, *SD* standard deviation, *CI* confidence intervals, *BMI* body mass index, *EDE-Q* Eating Disorder Examination—Questionnaire, *DASS-21* Depression Anxiety and Stress Scales, *STAI-S* Stress and Anxiety Inventory-State version, *MAAS* Mindfulness and Awareness Scale, *MEQ* Mindful Eating Questionnaire, *PFS* Power of Food Scale

**Table 3:** Means (and standard deviations) for the clinical outcome measures at post-intervention [8 weeks post-randomisation] and estimated within-group effect sizes (Cohen’s d with 95% confidence intervals) between baseline and post-intervention

Assessment	WL				MT				ABMT			
	*N*	Mean	SD	*d* (95% CI)	*N*	Mean	SD	*d* (95% CI)	*N*	Mean	SD	*d* (95% CI)
8-weeks post-randomisation heading missing												
BMI	11	33.73	5.51	0.27 (− 0.52 to 1.06)	15	32.68	4.54	0.07 (− 0.62 to 0.78)	13	34.48	8.61	0.03 (− 0.70 to 0.78)
Body fat percentage	11	38.51	14.10	− 0.31 (− 1.1 to 0.49)	14	39.05	12.43	0.06 (− 0.66 to 0.79)	10	40.76	9.19	− 0.19 (− 1.0 to 0.63)
EDE-Q Global	10	1.70	0.98	**0.90 (0.03 to 1.76)**	15	2.12	1.25	0.16 (− 0.56 to 0.88)	12	2.32	1.28	0.38 (− 0.38 to 1.15)
EDE-Q binge episodes	11	2.63	4.27	0.79 (− 0.04 to 1.62)	15	4.4	5.11	0.33 (− 0.37 to 1.04)	12	6.33	8.48	0.31 (− 0.45 to 1.0)
DASS-21 Depression	11	3.63	5.23	0.37 (− 0.42 to 1.17)	15	3.53	2.94	− 0.08 (− 0.78 to 0.61)	12	3.08	3.02	0.51 (− 0.26 to 1.27)
DASS-21 Anxiety	11	3.90	3.61	0.38 (− 0.41 to 1.18)	15	3.33	2.58	0.01 (− 0.68 to 0.72)	12	2.08	3.17	0.14 (− 0.61 to 0.90)
DASS-21 Stress	11	6.36	5.06	0.44 (− 0.35 to 1.24)	15	5.33	3.08	0.12 (− 0.58 to 0.82)	12	4.16	2.58	0.51 (− 0.26 to 1.27)
STAI-S	10	37.7	12.15	0.62 (− 0.23 to 1.46)	15	36.8	9.78	− 0.37 (− 1.09 to 0.35)	13	35.15	8.79	0.10 (− 0.63 to 0.84)
MASS	9	11.2	22.3	− 0.50 (− 1.3 to 0.38)	15	8.32	16.9	− 0.38 (− 1.11 to 0.35)	12	3.90	1.01	− 0.21 (− 0.97 to 0.54)
MEQ Global	4	2.66	0.36	− 0.18 (− 1.41 to 1.05)	11	2.52	0.34	− 0.96 (− 2.00 to 0.09)	8	2.50	0.58	− 0.33 (− 1.25 to 0.58)
MEQ-Awareness	10	2.82	0.49	− 0.08 (− 0.90 to 0.73)	14	2.49	0.55	0.00 (− 0.71 to 0.71)	12	2.38	0.68	− 0.27 (− 1.04 to 0.50)
MEQ-Distraction	9	2.74	0.66	− 0.31(− 1.17 to 0.56)	15	2.59	0.42	0.04 (− 0.66 to 0.74)	12	2.75	0.62	− 0.30 (− 1.09 to 0.48)
MEQ-Disinhibition	7	2.61	0.75	− 0.37 (− 1.36 to 0.62)	13	2.19	0.59	**− 0.90 (− 1.68 to − 0.11)**	10	2.25	0.79	− 0.18 (− 1.01 to 0.63)
MEQ-Emotional	10	2.77	0.93	− 0.25 (− 1.07 to 0.57)	13	2.63	0.54	− 0.69 (− 1.45 to 0.075)	12	2.58	0.70	− 0.41 (− 1.18 to 0.37)
MEQ-External	6	2.61	0.52	− 0.12 (− 1.10 to 0.85)	14	2.54	0.57	0.14 (− 0.72 to 1.01)	9	2.31	0.67	− 0.16 (− 0.96 to 0.63)
PFS	11	3.15	0.94	0.39 (− 0.40 to 1.18)	15	2.96	1.06	**0.82 (0.07 to 1.54)**	12	3.05	0.95	0.56 (− 0.21 to 1.33)
BOGUS test (g)	11	97.4	65.2	− 0.31 (− 1.10 to 0.48)	15	80.5	55.5	0.07 (− 0.62 to 0.78)	13	55.4	58.7	0.42 (− 0.32 to 1.17)

Bold font signifies that the CI do not include 0. *WL* waitlist, *MT* mindfulness training, *ABMT* Attention Bias Modification Training, *N* number, *SD* standard deviation, *CI* confidence intervals, *BMI* body mass index, *EDE-Q* Eating Disorder Examination – Questionnaire, *DASS-21* Depression Anxiety and Stress Scales, *STAI-S* Stress and Anxiety Inventory-State version, *MAAS* Mindfulness and Awareness Scale, *MEQ* Mindful Eating Questionnaire, *PFS* Power of Food Scale

#### Weight and body composition

Between- and within- group differences on BMI and body fat mass percentage were mostly small from baseline to post-intervention.

#### Eating behaviour

There was a medium size difference on the EDE-Q global scores between WL and MT (favouring WL), and all other between-group comparisons for this measure (including for EDE-Q objective binge eating episodes days) were of a small effect size. Within-group analyses on the global score of the EDE-Q showed a large size decrease (confidence intervals do not cross zero) in the WL group (*d* = 0.90, 95% CI 0.03 to 1.76). The number of days with objective binge eating episodes (OBEs) from baseline to post-intervention had a small to medium effect size reduction within all groups.

The total change score of the PFS (assessing psychological impact of food availability) from baseline to post-intervention showed a large effect size of *d* = 0.88 (95% CI 0.05 to 1.69) between WL and MT (confidence intervals not including zero), and a medium effect size between MT and ABMT, favouring MT in both cases.

Within-groups, there was a only a small effect reduction in PFS scores (lower scores indicate less hedonic hunger) from baseline to post-intervention in the WL group. This reduction was of a medium effect size for the ABMT group and of a large effect of *d* = 0.82 (95% CI 0.07 to 1.54) in the MT group (confidence intervals do not cross zero).

On a behavioural level, the change scores of food consumed (grams) during the Bogus taste test from baseline to post-intervention revealed a large effect size (confidence intervals do not include zero) between the WL and the ABMT groups (*d* = 0.87, 95% CI 0.02 to 1.71), favouring ABMT. However, the change on food consumption within groups (MT and ABMT) was of a small size.

#### Mood

Between-group effect sizes for mood symptoms ranged from small to large. The greatest difference was found in the state-anxiety scores measured by the STAI-S, showing a large effect size between WL and MT, favouring WL (*d* = − 0.77, 95% CI − 1.60 to 0.07). Within-groups effects sizes for mood outcomes ranged from small to medium in all groups.

#### Mindfulness

Effect sizes for dispositional mindfulness measured by the MAAS ranged from small to medium between-groups. Within-groups effect sizes showed a small to medium increase in MAAS scores across groups.

Global MEQ scores from baseline to post-intervention showed a large effect size in the difference between WL and MT (*d* = − 0.84, 95% CI − 2.31 to 0.69), favouring MT. Within both WL and ABMT, MEQ scores had a small increase from baseline to post-intervention and a large effect increase within the MT group.

### Secondary outcomes: baseline to follow-up (week 12)

Data on the clinical outcomes from baseline to follow-up are shown in Tables 4 and 5 for between-groups and within-groups respectively.

**Table 4:** Mean change scores (follow-up scores adjusted for baseline) for the clinical outcome measures at 12-week post-randomisation timepoints including the number of participants (N), means, and standard deviations (SD) for each trial arm, and the estimated between-groups effect size (Cohen’s *d* with 95% confidence intervals)

	MT			ABMT			Between- subject *d* (95% CI)
	*N*	Mean	SD	*N*	Mean	SD	MT versus ABMT
12-weeks post-randomisation (adjusted for baseline)							
BMI	12	− 2.83	8.15	11	− 1.16	1.49	0.27 (− 0.54 to 1.09)
EDE-Q Global	11	− 0.12	0.59	12	− 0.49	0.32	− 0.78 (− 1.62 to 0.07)
EDE-Q binge episodes	15	− 3.6	7.15	12	− 6.5	8.6	− 0.37 (− 1.13 to 0.39)
MEQ Global	5	0.39	0.24	6	0.23	0.28	− 0.60 (− 1.80 to 0.62)
MEQ-Awareness	14	0.32	0.59	10	0.28	0.56	− 0.07 (− 0.88 to 0.74)
MEQ-Distraction	15	0.08	0.80	10	0.23	0.47	0.20 (− 0.59 to 1.00)
MEQ-Disinhibition	15	0.69	0.46	8	0.32	0.31	− 0.87 (− 1.76 to 0.03)
MEQ-Emotional	14	0.5	0.5	11	0.25	0.62	− 0.44 (− 1.24 to 0.35)
MEQ-External	7	− 0.07	0.58	9	− 0.14	0.42	− 0.14 (− 1.13 to 0.84)
PFS	14	− 0.78	0.56	12	− 0.50	0.48	0.51 (− 0.27 to 1.29)

Follow-up scores minus baseline scores. Bold font signifies that the CI do not include 0. *WL* waitlist, *MT* mindfulness training, *ABMT* Attention Bias Modification Training, *N* number, *SD* standard deviation, *CI* confidence intervals, *BMI* body mass index, *EDE-Q* Eating Disorder Examination—Questionnaire, *DASS-21* Depression Anxiety and Stress Scales, *STAI-S* Stress and Anxiety Inventory-State version, *MAAS* Mindfulness and Awareness Scale, *MEQ* Mindful Eating Questionnaire, *PFS* Power of Food Scale

**Table 5:** Mean (and standard deviation) for the clinical outcome measures at post-intervention (12 weeks post-randomisation) and estimated within-group effect sizes (Cohen’s d with 95% confidence intervals) between baseline and follow-up

Assessment	MT				ABMT			
	*N*	Mean	SD	*d* (95% CI)	*N*	Mean	SD	*d* (95% CI)
12-weeks post-randomisation								
BMI	12	28.9	9.8	0.57 (− 0.19 to 1.33)	11	34.1	8.5	0.07 (− 0.70 to 0.84)
EDE-Q Global	13	2.24	1.3	0.04 (− 0.70 to 0.80)	12	2.34	1.38	0.36 (− 0.40 to 1.12)
EDE-Q binge episodes	15	3.4	3.8	0.33 (− 0.37 to 1.04)	12	3.33	5.6	0.75 (− 0.03 to 1.53)
MEQ Global	14	2.67	0.36	− **1.36 (**− **2.4 to **− **0.29)**	7	2.53	0.47	− 0.43 (− 1.38 to 0.52)
MEQ-Awareness	14	2.72	0.53	− 0.41 (− 1.13 to 0.31)	11	2.49	0.78	− 0.40 (− 1.20 to 0.39)
MEQ-Distraction	15	2.64	0.68	− 0.02 (− 0.73 to 0.67)	12	2.58	0.66	− 0.06 (− 0.84 to 0.72)
MEQ-Disinhibition	15	2.41	0.62	− **1.28 (**− **2.0 to **− **0.48)**	9	2.47	0.56	− 0.58 (− 1.44 to 0.29)
MEQ-Emotional	15	2.68	0.71	− 0.69 (− 1.42 to 0.04)	12	2.41	0.74	− 0.19 (− 0.96 to 0.58)
MEQ-External	15	2.72	0.63	− 0.15 (− 1.01 to 0.70)	11	2.31	0.38	0.40 (− 0.42 to 1.23)
PFS	14	2.97	0.78	**0.94 (0.18 to 1.7)**	12	3.0	0.94	− 0.21 (− 0.97 to 0.54)

Bold font signifies that the CI do not include 0. *WL *waitlist, *MT*  mindfulness training, *ABMT *attention bias modification training, *N* number, *SD* standard deviation, *CI* confidence intervals, *BMI* body mass index, *EDE-Q* Eating Disorder Examination—Questionnaire, *DASS-21* Depression Anxiety and Stress Scales, *STAI-S* Stress and Anxiety Inventory-State version, *MAAS* Mindfulness and Awareness Scale, *MEQ* Mindful Eating Questionnaire, *PFS* Power of Food Scale

### BMI

Between-group effect sizes for BMI change were small (baseline to follow-up). There was a medium size BMI reduction within the MT group.

#### Eating behaviour

For change in EDE-Q global scores (baseline to follow-up), the effect size between MT and ABMT was large (*d* = − 0.78, 95% CI − 1.62 to 0.07), favouring ABMT. The within-groups difference on EDE-Q global scores was of small effect. The number of days with OBEs (baseline to follow-up) had a small to medium effect size reduction within both groups.

The total change score of the PFS (baseline to follow-up) was of medium effect between MT and ABMT, favouring MT. Decreases in hedonic hunger remained as a large effect size (*d* = 0.94, 95% CI 0.18 to 1.7) within the MT group.

#### Mindfulness

Global MEQ scores (baseline to follow-up) showed a medium effect size in the difference between MT and ABMT, favouring MT, with higher levels of mindful eating scores at follow-up. There was a large increase (confidence intervals do not cross zero) in global mindful eating scores within the MT group (*d* = − 1.36, 95% CI − 2.4 to − 0.29), and a small effect increase within the ABMT group.

## Credibility and acceptability of training

Participants rated the credibility and usefulness of the training using VAS scales (0–100). The mean credibility score was 75.5 (*SD* = 13.3, *n* = 15) for MT and 71.3 (*SD* = 12.4, *n* = 12) for ABMT. The mean usefulness score was 70.1 (*SD* = 26.2, *n* = 15) for MT participants and 68.5 (*SD* = 18.3, *n* = 12) for ABMT. In terms of perceived benefits, 78.5% (*n* = 11/14) of MT participants and 81.8% (*n* = 9/11) of ABMT participants said they benefited from the training, with the remainder saying they did not. When asked whether they would recommend the training, 86.7% (13/15) of MT participants and 91.7% (*n* = 11/12) of ABMT participants said “yes”. When asked whether they would take up the training as a treatment if there was evidence of its benefits on eating habits and weight loss, 86.7% (13/15) of MT participants said “yes” compared to 100% of ABMT participants (*n* = 12/12). From people in the WL group, 54.5% (*n* = 6/11) opted for MT and 45.4% (*n* = 5/11) opted for ABMT after their 8-week waitlist period.

### Attention bias change: an exploration of potential mechanisms underpinning the interventions

We explored the effects of the interventions on AB change from baseline to post-intervention within the whole sample (*n* = 39). As an additional sensitivity analysis, and based on the rationale that attention trainings would modify initial AB to High-Cal food cues, we also assessed AB change in those with an initial attentional approach (i.e., AB scores greater than zero) to High-Cal food cues at baseline (i.e., “approach” subsample, n = 28). Results from the “approach” subsample mirrored the findings from the whole sample. Hence, only the whole sample data are described below. Means and standard deviations for all groups at baseline and post-intervention are shown in Additional file 2: Table S1).

#### Initial fixation duration bias for high caloric food

We found a significant interaction of time x group [*F*(2,36) = 7.68, *p* = 0.002, partial *η*^2^ = 0.299] (Additional file 1: Fig. S1.1) and a significant main effect of time [*F*(1,36) = 5.613, *p* = 0.023, *η*^2^ = 0.135], associated effect sizes were large. No main effect of group was found [*F*(2,36) = 0.207, *p* = 0.814, *η*^2^ = 0.011], and the associated effect size was small.

As a *post-hoc* analysis, we used paired-samples T-tests to look at the change of initial fixation duration bias to High-Cal food at baseline versus post-intervention within each group. Results showed a significant mean difference of 459.08 ms (95% CI 123.59 to 794.57) from baseline to post-intervention within the ABMT group [*t*(12) = 2.981, *p* = 0.011, *d* = 0.82] with a large effect size, indicating a reduction in AB to High-Cal food cues. However, no significant differences were found within the MT group, with a mean difference from baseline to post-intervention of − 1.47 ms (95% CI − 106.92 to 103.98), [*t*(14) = − 0.030, *p* = 0.977, *d* = 0.00]. As expected, the mean difference from baseline to post-intervention within the WL condition [− 44.03 ms (95% CI − 189.94 to 101.86)] was non-significant [*t*(10) = − 0.673, *p* = 0.516, *d* = − 0.20], associated effect sizes were small.

#### Gaze duration bias to high caloric food

We found a significant time x group interaction [*F*(2,36) = 8.619, *p* > 0.001, *η*^2^ = 0.324] (Additional file 1: Fig. S1.2), and a main effect of time [*F*(1,36) = 6.936, *p* = 0.012, *η*^2^ = 0.162], both associated effect sizes were large. No main effect of group was found [*F*(2,36) = 0.697, *p* = 0.505, *η*^2^ = 0.037] with a small effect size.

A *post-hoc* analysis into the mean differences from baseline to post-intervention within groups in the whole sample revealed a significant mean difference in the ABMT group of 640.29 ms (95% CI 227.34 to 1053.24), [*t*(12) = 3.378, *p* = 0.005, *d* = 0.93] with a large associated effect size, revealing a significant decrease in AB to High-Cal food cues. The mean difference within the MT group [− 27.39 ms (95% CI − 211.98 to 157.19)] was non-significant [*t*(14) = − 0.318, *p* = 0.755, *d* = − 0.08]. Similarly, the mean difference from baseline to post-intervention within the WL group was non-significant [− 14.00 ms (95% CI − 201.09 to 173.07)], [*t*(10) = − 0.167, *p* = 0.871, *d* = 0.05], both associated effect sizes were small.

#### AB change and OBEs

Based on the reduction in AB to High-Cal food cues within the ABMT group, an exploration of the correlational relationship between change in initial fixation duration bias to High-Cal food cues and change in days with OBEs (EDE-Q, item 15) was made. Analyses revealed a significant correlation between change in AB to High-Cal food cues and change in days with OBEs in the ABMT group [*r*(11) = − 0.68, *p* = 0.01], i.e., the greater the reduction on attentional approach to High-Cal food cues, the greater the reduction of OBEs at post-intervention. No correlations between AB change and days with OBEs were found in the MT or WL groups.

## Discussion

The main feasibility aim of this study was to establish recruitment and retention rates, and treatment adherence. Our findings suggest that the protocol is highly feasible. We were able to recruit the intended number of participants in a timely manner: the main reasons why people who showed interest did not participate were related to time commitment and the lack of monetary compensation for travel. Retention rates from baseline to follow-up were higher than expected (> 75%) and treatment session completion was high in both groups. However, adherence to home training practice was different between groups, with those in the ABMT group only completing a third of recommended home practice, whereas on average, MT participants completed all home sessions. These data are in accord with reports regarding threat-avoidance trainings for people with anxiety [24, 61]. Poorer compliance with ABMT may be related to its repetitive nature which could be improved by creating a more engaging version [62].

Regarding the secondary aims of this study, we calculated both, between-group and within-group effect sizes for our clinical outcomes. For BMI and body fat percentage, between- and within-groups effect sizes were mostly small, except for the baseline to follow-up BMI change within the MT group, where there was a medium size reduction. This preliminary indication of the effect of MT on BMI is in line with meta-analyses of MBIs in obesity showing that those receiving MBIs, continue to lose weight at follow-up, compared to those in control groups [9, 46]. The effects of ABMT on BMI are largely unknown. A small uncontrolled multi-session study of food-related ABMT, found significant reductions in BMI after 8 weeks of training in overweight or obese binge eaters [31]). However, they did not find a reduction in AB to food cues, and given the lack of a control group, the reductions in BMI could be a placebo effect [63], or a fluctuation over time [64].

We found large between-group effect sizes in eating disorder symptoms (EDE-Q global scores) from baseline to follow-up between trainings (i.e., MT vs.ABMT), with larger EDE-Q score reductions in the ABMT group. This suggests that in people with obesity ABMT may have more potential for reducing eating disorder symptoms, than MT. This is line with Boutelle et al. [31] who found significant reductions in global EDE-Q scores, as well as fewer days with OBEs in response to an ABMT intervention. Another small trial used a version of the anti-saccade task in people with BED for inhibition rather than attention training and, found significant post-training reductions in OBEs [65]. However, they found a similar reduction in OBEs in the control condition (sham training). Likewise, participants in our WL group also had a large reduction in eating disorder symptoms from baseline to post-intervention. It is possible that participants in the WL group were very motivated to change their eating behaviour, which may have led to improvements whilst waiting.

Based on global change scores from the PFS, there were greater reductions in hedonic hunger in the MT group which lasted until follow-up. This was evidenced by a large between-group effect between MT and WL from baseline to post-intervention and a medium effect between MT and ABMT. In addition, within-group effects on PFS scores were large in MT. Hedonic hunger has been correlated with other aspects of problematic eating such as emotional eating and disinhibited eating. These have been proposed to be part “food reward responsivity”, which may contribute to the development/maintenance of obesity [66].

In relation to food consumption (Bogus taste test), there was a large between-group effect size in the comparison between ABMT and WL, with ABMT participants showing a greater reduction in the food consumed at post-intervention. However, within-group effects in ABMT were small. Other studies investigating the effects of ABMT on eating behaviour have also reported a decrease in food consumption after training. However, in some studies the taste test consisted of the same item used for the training, which limits generalisability of findings [15, 19]. In contrast, studies using different types of foods in the taste test to those used in the training, have reported reduced High-Cal food consumption after the intervention [67, 68]. However, due to study design (single session trainings), these studies did not include a baseline measure of food consumption. Unlike other studies [67, 68], we only included High-Cal foods in the Bogus taste test. Including low caloric foods in addition to High-Cal ones would have given individuals the option to make a healthier choice from baseline to post-intervention. Furthermore, it is possible that due to social desirability bias [69], individuals consumed a smaller amount of food during the test compared to a more naturalistic setting, leading to a floor effect across groups. Lastly, we did not measure the effects of training accuracy rates. Werthmann et al. [15] showed that reduced food consumption post-training only occurred in those with high accuracy scores during the training.

Global mindful eating scores (measured by the MEQ) in the MT group revealed medium to large between- and within-group effect sizes from baseline to post-intervention, and baseline to follow-up. This is supported by studies using mindful-eating interventions (see [70] for review). Similarly, the disinhibition, and emotional eating subscales of the MEQ revealed medium to large effect sizes between- and within-groups at both timepoints. The MT within-group effect in the MEQ-disinhibition subscale was greater at follow-up suggesting a more durable effect of the MT training that can strengthen with time. Consistent with this idea, one RCT using Mindfulness-based Eating Awareness Training found that improvements in clinical outcomes were sustained (and in some cases improved) at a 4-month follow-up [21].

To explore potential mechanisms, we investigated the effects of each intervention on AB to High-Cal food cues at post- intervention. Those in the ABMT showed a significant reduction in AB to High-Cal food cues (i.e., less approach) at post-intervention. However, those in the MT and WL groups did not change. Changes in AB in those in the ABMT group are in line with what was predicted. Previous research indicates that single-session ABMT can influence attention in the trained direction (e.g., training to direct attention towards chocolate increases attention bias for chocolate). [14, 15, 19, 30]. Similarly, training attention towards Low-Cal food can reduce attention to High-Cal foods [67, 68]., and increase attention towards Low-Cal food [30]. It is worth noting that the observed reduction in AB to High-Cal food within the ABMT group was mainly driven by in-person sessions (given that home training completion rates for this group were very low). This suggests that mixed training delivery methods (i.e., home practice in addition to in-person sessions) may not be necessary. With the MT training, our results are not in accord with what has been reported in studies using MBIs in addiction and chronic pain [71]. However, in addition to the differing measurement of AB (RTs vs. eye movements), the above studies used a more complex form of MBI including different treatment components, whereas our MT involved only brief meditations related to mindful eating and cravings. These differences could partly explain the variations in AB change.

In relation to the observed changes in AB to High-Cal food cues at post-intervention within the ABMT group, correlation analyses revealed a significant association between reductions in attentional approach and reductions in OBE days at post-intervention in the ABMT group. This is in line with findings from other studies [31, 65] and highlights the potential of ABMT to reduce eating disorder psychopathology.

Our data suggest that ABMT may modify disordered eating behaviour via changes in AB to food, and therefore improving relatively automatic cognitive-control processes. In contrast, MBIs may target a different level of attention processes (i.e., less automatic than AB). We found that MT reduces emotional eating and disinhibition (i.e., measured by the MEQ subscales) together with hedonic hunger, suggesting that MBIs may involve strengthening cognitive-control processes, which improves emotion regulation and lowers impulsivity-related behaviours [72].

Limitations of this feasibility study include its small group size and generalisability of findings to wider population of people with overweight or obesity (most participants were younger females). BMI ranged from overweight to class 3 obesity and some participants reported ED symptoms: these differences could have affected our results based on different cognitive and social characteristics that might interact with the effects of the trainings. Future studies should conduct sensitivity analyses to identify any differences between individuals with and without ED symptoms, and across different BMI categories. We had a relatively short follow-up period for a trial investigating rather complex behavioural and cognitive patterns, which questions the sustainability of the observed changes (particularly in the absence of further ABMT/MT training). In addition, given that our follow-up measure was done online, we did not include AB assessment. However, an online follow-up (as opposed to an in-person assessment) potentially leads to higher retention rates. Lastly, we did not assess our WL group at follow-up to establish whether early improvements in that group persisted or were short-lived.

## Conclusions and future directions

Our findings indicate that both ABMT and MT may have value as adjunctive treatments of obesity and BED, but that their underlying mechanisms may differ. Results indicate that it is feasible to conduct a larger scale RCT comparing ABMT with MT and a WL control group. Both interventions were credible, acceptable, and had high retention rates. A future trial should remedy differences in home practice completion rates. Both trainings are low-cost, easy to administer and can be done online.

## Supplementary Information

**Additional file 1. Supplementary Figure S1.1.** Mean initial fixation duration bias (and SEs) for high caloric food cues at baseline (timepoint 1) and post-intervention (timepoint 2) comparing the WL, ABMT and MT conditions in the whole sample. **Supplementary Figure S1.2** Mean duration bias (and SEs) for high caloric food cues at baseline and post-intervention comparing the WL, ABMT and MT conditions in the whole sample.

**Additional file 2. Supplementary Table S1:** Mean scores and standard deviations for the different measures of attention bias in the ABMT, MT and WL groups in the whole sample before and after the intervention.

## Data Availability

The datasets used and/or analysed during the current study are available from the corresponding author on reasonable request.

## Abbreviations

AN: Anorexia nervosa
ACC: Anterior cingulate cortex
AB: Attention bias
ABMT: Attention bias modification training
BED: Binge eating disorder
BMI: Body mass index
BN: Bulimia nervosa
CI: Confidence intervals
CONSORT: Consolidated Standards of Reporting Trials
DASS: Depression, Anxiety, Stress scale
ED: Eating disorder
EDE-Q: Eating Disorder Examination Questionnaire
ES: Effect size
High-Cal: High caloric
MEQ: Mindful Eating Questionnaire
MAAS: Mindfulness Attention and Awareness Scale
MBIs: Mindfulness-based interventions
MT: Mindfulness training
N: Number of observations
OBE: Objective binge eating episodes
OSFED: Other specified feeding or eating disorder
PFS: Power of Food Scale
RCT: Randomised controlled trial
STAI: State and Trait Anxiety Inventory
SD: Standard deviation
WL: Waitlist
