# Supplementary material for: A randomised controlled feasibility study of food-related computerised attention training versus mindfulness training and waiting-list control for adults with overweight or obesity: the FOCUS study
Source: J Eat Disord. 2023 Apr 12;11:61. doi: 10.1186/s40337-023-00780-5 (PMC10099893; doi:10.1186/s40337-023-00780-5)
Supplement: Supplementary file 1 — Additional file 1. Supplementary Figure S1.1. Mean initial fixation duration bias (and SEs) for high caloric food cues at baseline (timepoint 1) and post-intervention (timepoint 2) comparing the WL, ABMT and MT conditions in the whole sample. Supplementary Figure S1.2 Mean duration bias (and SEs) for high caloric food cues at baseline and post-intervention comparing the WL, ABMT and MT conditions in the whole sample. [file 40337_2023_780_MOESM1_ESM.docx]

**Supplementary Figure S1.1** **Mean initial fixation duration bias (and *SE*s) for high caloric food cues at baseline (timepoint 1) and post-intervention (timepoint 2) comparing the WL, ABMT and MT conditions in the whole sample.**

**Supplementary Figure S1.2** **Mean duration bias (and *SE*s) for high caloric food cues at baseline and post-intervention comparing the WL, ABMT and MT conditions in the whole sample.**
