# Supplementary material for: A randomised controlled feasibility study of food-related computerised attention training versus mindfulness training and waiting-list control for adults with overweight or obesity: the FOCUS study
Source: J Eat Disord. 2023 Apr 12;11:61. doi: 10.1186/s40337-023-00780-5 (PMC10099893; doi:10.1186/s40337-023-00780-5)
Supplement: Supplementary file 2 — Additional file 2. Supplementary Table S1: Mean scores and standard deviations for the different measures of attention bias in the ABMT, MT and WL groups in the whole sample before and after the intervention. [file 40337_2023_780_MOESM2_ESM.docx]

**Supplementary Table S1**: **Mean scores and standard deviations for the different measures of attention bias in the ABMT, MT and WL groups in the whole sample before and after the intervention**

|  | General (food vs non-food) | | | | | | High caloric food | | | | | | Low caloric food | | | | | |
| --- | --- | --- | --- | --- | --- | --- | --- | --- | --- | --- | --- | --- | --- | --- | --- | --- | --- | --- |
|  | ABMT  (n=13) | | MT  (n=15) | | WL  (n=11) | | ABMT  (n=13) | | MT  (n=15) | | WL  (n=11) | | ABMT  (n=13) | | MT  (n=15) | | WL  (n=11) | |
|  | T1 | T2 | T1 | T2 | T1 | T2 | T1 | T2 | T1 | T2 | T1 | T2 | T1 | T2 | T1 | T2 | T1 | T2 |
| Direction bias (%)  (mean ±SD) | 52.7  (6.2) | 49.8  (4.0) | 49.3  (3.8) | 49.8  (2.8) | 54.0  (4.6) | 53.1  (2.5) | 54.4  (7.6) | 51.0  (4.9) | 52.4  (4.0) | 52.1  (7.6) | 63.0  (31.4) | 54.7  (4.5) | 51.1  (6.11) | 48.7  (8.0) | 46.3  (6.4) | 47.5  (4.1) | 42.3  (39.5) | 51.5  (6.1) |
| Initial fixation bias (ms)  (mean ±SD) | 201.4  (230.9) | -124.5  (222.2) | 33.3  (149.0) | 36.9  (169.3) | 45.6  (132.9) | 107.3  (90.6) | 281.7  (343.3) | -177.2  (402.5) | 57.4  (200.8) | 58.8  (240.1) | 79.0  (192.0) | 123.1  (121.6) | 110.2  (179.0) | -62.5  (275.2) | 18.3  (220.3) | 9.3  (122.7) | 12.5  (102.5) | 105.3  (152.3) |
| Duration bias (ms)  (mean ±SD) | 234.1  (309.9) | -93.1  (226.0) | 100.6  (218.4) | 86.7  (228.1) | 68.7  (202.7) | 149.3  (108.3) | 324.5  (406.8) | -315.7  (657.3) | 118.2  (301.7) | 145.6  (355.0) | 131.3  (314.4) | 145.3  (193.2) | 143.7  (287.9) | 129.5  (431.1) | 83.0  (213.3) | 27.8  (159.5) | 6.2  (200.7) | 153.2  (159.7) |
